# The next generation of Rwandan physicians with a primary health care mindset

**DOI:** 10.4102/phcfm.v7i1.885

**Published:** 2015-07-11

**Authors:** Maaike Flinkenflögel, Patrick Kyamanywa, Vincent K. Cubaka, Phil Cotton

**Affiliations:** 1 College of Medicine and Health Sciences, University of Rwanda, Rwanda; 2 Inshuti Mu Buzima/Partners In Health, Rwinkwavu, Rwanda; 3 Department of Public Health, Aarhus University, Denmark

Globally there is a need for well-trained primary health care physicians at the district level. Physicians who focus on ambulatory care will be in greater demand in addressing the global burden of chronic disease and multi-morbidity, which are on the increase in Africa. Not surprisingly, family medicine has grown stronger on the African continent in the past decades.^1,2,3^ In Rwanda, education of health professionals has recently undergone several changes. Postgraduate training in medical and surgical specialties has been further developed in a constructive and inclusive way with support of American universities.^4^ Although postgraduate training in family and community medicine has been temporarily halted, the need to develop and enhance undergraduate training in social and community medicine was identified and efforts have since commenced. This raises the question whether postgraduate training was developed too early, at a time when undergraduate training did not yet embrace the concept of primary health care.

Rwanda is a small, landlocked, low-income country in central East Africa and the most densely populated country on the continent.^5^ The majority of the population live in rural areas. The 2010 Demographic Health Survey showed dramatic gains in life expectancy, although infectious diseases were still the leading cause of death.^6^ Chronic non-communicable diseases are on the rise and presently constitute 25% of Rwanda's burden of disease, which makes the need for chronic care urgent.^7^

At present Rwanda has four national referral hospitals, over 40 district hospitals and about 450 health centres. In addition, approximately 45 000 community health workers provide health promotion activities as well as preventive and curative care to rural communities.^8,9^ Faced with a similar shortage of health workers as in other African countries, Rwanda has successfully embarked on pre-service and in-service training programmes at all levels of care delivery.

## History of family and community medicine training in Rwanda

In 2008, the then National University of Rwanda (now University of Rwanda) started the postgraduate family and community medicine (FAMCO) training programme. The objective was to train family physicians capable of providing comprehensive, continuous health care of good quality, which is person centred, family oriented and community based, using the biopsychosocial model of care at the district level.^10^ Rwanda already had a robust structure and organisation in primary care through a network of community health worker teams and health centres. The FAMCO department, working with external partners, saw this as an opportunity for preparing a generation of well-trained, expert generalists able to work at the district level. However, by 2011 the programme was facing difficulties owing to stakeholders, from undergraduate students to policy makers, being unfamiliar with the new model of ‘African family medicine’. Faced with a national need to focus resources on training hospital-based specialists, the FAMCO residency programme was suspended in 2012.

## Training undergraduate medical students in community medicine

When the postgraduate training started in 2008, primary health care was not part of the undergraduate medical curriculum, except for a module in public health. Students had little exposure to district health care. In 2011, the FAMCO department developed a programme aimed at teaching community medicine as part of the undergraduate curriculum. The training consisted of a two-week theoretical module in the fourth year and a four-week practical module in the fifth year. The objective of this training was to prepare students, in small groups, to understand the unique issues and challenges of health in the community and medical care at the community level through experience at community health centres and district hospitals to enable them to function more effectively in the Rwandan health system.^11^ Both training content and teaching methods, which involved peer education, reflection and mentorship, were highly appreciated by the students.

During their review of the undergraduate curriculum in 2014, the Discipline of Primary Health Care (formerly the FAMCO department) advocated for students to be exposed to the concepts of social and community medicine earlier. In the emergent five-year curriculum, a new approach to social and community medicine (referred to as iSOCO) was developed and integrated from the first year of training. According to this training approach, students are taught in a multidisciplinary way (together with pharmacy and dentistry students) using the flipped classroom model,^12^ which includes e-learning methods. iSOCO aims to prepare each future Rwandan medical doctor to be patient centred and community oriented, to provide health care in a biopsychosocial way, to understand the importance of and having skills in communication, collaboration, advocacy, management, being a scholar and developing a professional attitude.^13^ Graduates will also appreciate the role of clinical information systems, decision support systems and referral pathways, the virtues of planned and coordinated care, and the role of self-management through exploration and appreciation of commonly held health beliefs.

## Conclusion

The new curriculum will use the biopsychosocial approach to develop a new generation of Rwandan doctors who are patient-centred and community-oriented change agents and are able to practise interpretive medicine and support patients to maintain their health. These attributes are described as the making of the desired Rwandan doctor.^13^ These doctors will be able to use the principles of primary health care in later medical or surgical specialisation or other career development paths.

To train postgraduate residents in primary health care and family medicine, it is essential to create awareness already at the undergraduate level so that there will be a critical mass of physicians who are able to apply these principles in their daily work at any level of the health care system. The new approach will prepare physicians to work in the primary health care setting to serve the needs of the Rwandan community and to mitigate the burden of disease at a lower level of care.
